# Correlation of the P300 evoked potential in depressive and cognitive aspects of aging

**DOI:** 10.5935/1808-8694.20120013

**Published:** 2015-11-20

**Authors:** Elisiane Crestani de Miranda, Maria Madalena Canina Pinheiro, Liliane Desgualdo Pereira, Maria Cecília Martineli Iorio

**Affiliations:** a PhD in Sciences - UNIFESP; Professor of Speech and Hearing Therapy - School of Medical Sciences - Santa Casa de São Paulo (FCMSCSP); b PhD in Sciences - UNIFESP; Professor of Speech and Hearing Therapy - UFSC; c Senior Associate Professor - UNIFESP; Professor of Speech and Hearing Therapy - UNIFESP; d Senior Associate Professor - UNIFESP; Head of the Speech and Hearing Therapy - UNIFESP). Departamento de Fonoaudiologia da Universidade Federal de São Paulo - UNIFESP

**Keywords:** aging, cognition, depression, event-related potentials, P300, hearing loss

## Abstract

The P300 is a long-latency auditory evoked potential highly dependent on cognitive skills. It is believed that cognitive changes caused or not by depressive symptoms may interfere with the P300.

**Aim:**

To investigate the influence of aging, cognitive and depression aspects of the P300 latency in elderly people.

**Methods:**

Clinical and experimental study with 60 elderly patients with sensorineural hearing loss of mild to moderately severe level, 20 males and 40 females, average age of 71.1. Participants were submitted to the long latency auditory evoked potential, in which the P300 latency (milliseconds) was studied. The cognitive aspects were assessed using the Mini-Mental State Examination (MMSE) and Alzheimer's Disease Assessment Scale (ADAS-Cog). In the assessment of depressive symptoms the Geriatric Depression Scale (GDS-15) was applied.

**Results:**

We found a significant positive correlation between latency and age (*p* = 0.031). There were no significant differences among the P300 latency and the ADAS-Cog (*p* = 0.584), MMSE (*p* = 0.199) and GDS (*p* = 0.541) categories.

**Conclusion:**

Aging caused an increase in the P300 latency; however, cognitive performance and the presence of depressive symptoms did not influence the P300 results in this elderly population.

## INTRODUCTION

The higher population longevity brings about an increase in organic, functional and psychosocial diseases and dysfunctions to the elderly. Among these, there are deterioration of the auditory function, cognitive decline and depression symptoms - situations that can worsen the aging process.

With the body's aging, many structural changes affect the auditory nerve along the brainstem and temporal lobe auditory pathways^1^. The effects of aging on the peripheral and central auditory systems interact with the cognitive decline, and hearing loss may worsen the effects of a cognitive deficit and vice-versa^2^, ^3^, ^4^.

These changes stemming from the aging process may lead to a number of changes in family and social lives. Among such changes is the onset or worsening of depression symptoms, because of communication difficulties generated by a reduction in hearing^5^^,^^6^ Another relevant fact is that depression may simulate dementia, for it worsens the performance of patients in cognitive tests^7^.

Elderly with depression may have important functional and cognitive changes. Many of these changes are similar to the ones seen in dementia, while others are more similar to normal aging. The most frequent cognitive changes in depressed elderly are the executive functions, attention deficits and reduction in processing speed^8^.

The P300 Long Latency Auditory Evoked Potentials reflect the functional use the individual makes of the auditory stimulus, and among the evoked auditory potentials this is the one which better reflects mental functioning, being highly dependent on cognitive skills, i.e. attention and discrimination^9^^,^^10^.

Aging is known for degenerating brainstem structures, and as a consequence, it increases the absolute latencies of auditory evoked potentials^1^^,^^11^, ^12^, ^13^. Even when considered an objective assessment method, the P300 may suffer interference from other factors which contribute to the variability of its measurements. Researchers Tremblay & Burkard^14^ discussed about the hard task of establishing homogeneous criteria for this potential in studies involving the elderly, because such population also suffers intrinsic and extrinsic influences of the very aging process. When examining this population, hardly ever one finds completely healthy individuals and, for this reason, sometimes there are paradoxical findings in the studies encompassing the population at this age range.

Aiming at having better safety in the clinical and scientific use of the P300 in the elderly, it is necessary to investigate the influence of some factors which are commonly seen in the population at this age range, such as cognitive decline and depression symptoms. The hypothesis used as basis for the present study was that cognitive changes - whether generated or not by depression symptoms - may impact the P300 cognitive potential. Thus, this study aims at investigating the influence of aging, cognitive aspects and depression symptoms in the P300 latency in elderly with hearing loss.

## METHOD

### Ethical concerns

This study is an experimental research with a non-probabilistic sample for convenience purposes; and it was approved by the Ethics in Research Committee of our Institution, under protocol # CEP 09/0149. The subjects were informed about the goals and methodology of the study and agreed to participate freely, signing the Consent Form.

### Sample selection and series

We considered the following eligibility criteria to make up the sample: a) having symmetrical mild to moderately-severe bilateral sensorineural hearing loss^15^; c) not having overt neurological and/or psychiatric diseases; d) not use any medication or drug which acts in the central nervous system that could alter the person's concentration or attention and e) being 60 years old or older, in other words, be an elderly according to the National Statute of the Elderly (Brazil, 2003).

The elderly which made up the sample were randomly selected in a clinic for the hearing impaired of a public hospital, after an initial analysis of their charts to check whether they met the aforementioned inclusion criteria.

Thus, the sample had 60 elderly with mild to moderately-severe sensorineural hearing loss, 40 (66.7%) females and 20 (33.3%) males, in the age range between 61 and 85 years (mean of 71.7 years - SD-6.1) and mean schooling of 5.4 years (SD-4.2).

### Procedures

Initially, all the elderly were submitted to assessment of their cognitive and psychological aspects in order to classify their performances as normal or abnormal. Following that, they were submitted to electrophysiological assessment. These procedures were carried out in two sessions, in the first we employed the cognitive and psychological assessment protocols, in the second the subjects were submitted to electrophysiological testing.

### Assessment of cognitive aspects

All the participants were submitted to cognitive assessment by means of the Mini-Mental Exam (MME) and the Alzheimer's disease Assessment Scale - ADAS - Cog.

The MME is a tracking test, used to assess the cognitive function, in Brazil it was adapted by Bertolucci et al.^16^. The test assesses eight cognitive parameters, broken down into seven categories: temporal and spatial orientation, short-term memory and evoking of words, calculus, praxis, language and visual-spatial skills. The test score varies between 0 and 30 points. The lower the score, the greater the likelihood of the subject having cognitive abnormalities. In this study we used the score classification according to Brucki et al.^17^.

The cognitive session of the Alzheimer's Disease Assessment Scale (ADAS-Cog) is made up of 11 items, which assesses memory (recognition and evocation), language (discourse and understanding) and praxis (copy and ideomotor). The score varies from 70 (lowest) to zero. The greater the score, the greater is the cognitive involvement in the assessed individual. The score was classified according to the individual's education and according to the classification criteria of the mean plus two standard deviations - according to Schultz et al.^18^.

We should stress that this scale was used solely to classify the cognitive performance of the individuals in the normal or abnormal categories, according to their years of schooling. None of the participants was diagnosed with Alzheimer's Disease. The use of these scales took around 50 minutes.

### Assessing depression-related aspects

To assess depression symptoms we employed the abridged version of the Geriatric Depression Scale (GDS-15), made up by 15 questions with closed answers options (“yes” or “no”). To analyze the results we considered the score criteria suggested by Almeida & Almeida^19^, and scores higher than five points were considered abnormal. Deployment time was five minutes.

### P300 - electrophysiological assessment

In order to assess the P300 Long Latency Auditory Evoked potential we used the four-channel Biologic Systems Corp. equipment, with the Evoked Potential System version 5.7, Model 92, and a Pentium R microcomputer. The exam was carried out in a silent and semi-dark room. The individuals were awake, sitting in a comfortable chair.

The individual was first asked concerning the use of medication in the 24 hours prior to the exam, doing extraneous mental or physical activities, smoking and/ or ingestion of stimulants, i.e. tea, coffee or chocolate. All the participants answered “no” to these questions.

Electrode positioning followed the International Electrode System (IES) 10-20 (Jasper^20^) standard, namely, in the front, (Fz) the ground electrode, in the midline of the cranial vertex, (Cz) the active electrode, and in the ear lobes (A1 = left ear and A2 = right ear) the reference electrode.

We used a tone burst stimulus in the frequencies of 500 and 1000 Hz, since these are the ones more frequently preserved in individuals with hearing loss. The stimuli were presented in an odd-ball type of paradigm (20%-80%), the rare tone burst was in the 1000 Hz frequency, and the other stimulus - the frequent tone burst was in the 500 Hz frequency. The patient was instructed to pay attention to the different stimuli (rare stimulus) which appeared randomly, in a sequence of equal stimuli (frequent stimulus).

The stimuli were presented in only one ear by means of an insertion ER-3A earbud. The stimuli presentation level varied between 70 and 88 dBHL, according to the hearing threshold for pure tones without a hearing aid. Each stimulus lasted for 200 ms, and the rise-decay was 50 ms. There were 300 stimuli in the scan. The stimuli presentation speed was 1.1/s with alternate polarity, and the recording window was of 512 ms.

In this study, the latency was marked only in the rare stimulus trace at the maximum point of the P300 wave amplitude, after the N1-P2-N2 complex. The individuals who did not have responses for the P300 were represented with the maximum latency for the recording, corresponding to 500 ms.

### Statistical method

Initially, we investigated the agreement between the latencies on both ears, considering the intraclass correlation coefficient (ICC)^21^. We used the Spearman Correlation Coefficient^®^ as a correlation measure for the Latency with Age and Education^22^. The Mann-Whitney test was employed to compare the latency mean value with the degree of hearing loss, as well as to compare the P300 distributions in the ADAS-cog, MME and GDS response category. In each hypothesis test, the level of significance was set in 0.05.

## RESULTS

In the sample studied, there was a high occurrence of abnormalities in the cognitive tests; nonetheless, depression symptoms were not very much present in this group, according to what is depicted on Table 1.

**Table 1:** Distribution of the ADAS-Cog, MME and GDS frequencies and percentages.

	Abnormal		Normal		Total	
Test	n	%	n	%	n	%
ADAS	30	50.0%	30	50.0%	60	100.0%
MME	37	61.7%	23	38.3%	60	100.0%
EDG	9	15.0%	51	85.0%	60	100.0%

The mean latency value in the right ear was 359.6 ms (SD = 52.8) and the left ear was 358.8 ms (SD 53.3). There was a strong agreement between the latencies from both ears, which Intraclass Correlation Coefficient value (ICC_o_) was equal to 0.97 and the 95% confidence interval for the ICC of [0.95 to 0.98]. Continuing with the P300 latency analysis, we considered the mean values from the observations in the two ears.

We noticed that the education variable did not influence the P300 latency; nonetheless, there was a positive correlation between the P300 latency and age - Table 2.

**Table 2:** P300 (ms) latency Spearman correlation coefficients with age and schooling.

	Correlation coefficients
Age	r = 0.279 (*p* = 0.031*)
Schooling	r = −0.024 (*p =* 0.854)

r: correlation coefficient of Spearman;

* significant positive correlation.

On Table 3 we notice that as age increases, there is a tendency towards increasing the P300 latency (ms).

**Table 3:** Latency-descriptive statistics for latency (ms) per age range.

Age range	n	Mean	Standard deviation	Lowest	Median	Highest
60-69	24	342.1	45.0	289.2	337.0	500.0
70-79	29	368.9	58.1	272.7	358.2	500.0
80 ou +	7	377.6	42.2	305.7	387.7	422.7
Total	60	359.2	52.7	272.7	348.0	500.0

The P300 latency was also analyzed according to the hearing loss degree mean value in the frequencies of 500 and 4000 Hz. We initially had a group of individuals with a mean value of 41 to 50 dBHL in the 500 to 4000 Hz frequency range (mean of 45.2 dB) and a second group formed by individuals with means of 51 to 70 dBHL in the 500 to 4000 Hz frequency range (mean of 55.9 dB). We noticed that there was no influence of the degree of hearing loss in the P300 latency - Table 4.

**Table 4:** P300 latency (ms) descriptive statistics according to the hearing loss degree variable, per ear.

Ear	Threshold	n	Mean	Standard deviation	Lowest	Median	Highest
	Up to 50 dB	30	360.6	57.6	279.2	350.2	500
RE	> 50 dB	30	358.7	48.4	291.2	348.2	500
	Total	60	359.6	52.8	279.2	348.2	500
	Up to 50 dB	30	358.1	57.7	266.2	341.7	500
LE	> 50 dB	30	359.4	49.9	287.2	352.7	500
	Total	60	358.8	53.4	266.2	346.7	500

Mann-Whitney test. P300 latency mean × Degree of HL. *p:* 0.862. LE: left ear; RE: right ear.

On Table 5 we can see the descriptive statistic values for the P300 latency in each category of results (normal and abnormal) from the ADAS-cog, MME and GDS. We did not find significant differences between the P300 distributions in the two ADAS-cog categories. The same happens in the two MME categories and the two GDS categories. Therefore, there are no associations between the P300 with the ADAS-cog, MME and GDS.

**Table 5:** P300 latency descriptive statistics in each category of the ADAS-cog, MME and GDS.

Test	Categories	n	Mean	Standard deviation	Lowest	Median	Highest
	Abnormal	30	361.2	54.5	272.7	350.2	500.0
ADAS-Cog	Normal	30	357.2	51.8	293.2	340.5	500.0
	Total	60	359.2	52.7	272.7	348.0	500.0
	Abnormal	37	368.1	59.9	272.7	355.2	500.0
MME	Normal	23	344.8	35.0	293.2	340.2	414.7
	Total	60	359.2	52.7	272.7	348.0	500.0
	Abnormal	9	359.3	38.8	293.7	373.2	397.7
GDS	Normal	51	359.2	55.1	272.7	345.7	500.0
	Total	60	359.2	52.7	272.7	348.0	500.0

Mann-Whitney test. P300 mean latency × ADAS-Cog p: 0.584. P300 mean latency X MME p: 0.199. P300 mean latency X GDS p: 0.541.

## DISCUSSION

Among the diseases and dysfunctions which affect the elderly population, the drop in hearing is one of the most frequent. In this age range, besides the hearing loss, it is common to have a drop in cognition. Many of the hearing loss symptoms are similar to dementia symptoms, and both result in communication difficulties. Thus, often times in the beginning, cognitive disorders may be unnoticed among the elderly with presbycusis.

In the two cognitive tests employed, the score was low comparing with the mean education of 5.4 years in our sample. Results show a high rate of cognitive changes in this group, which supports the hypothesis that the hearing loss may contribute to cognitive dysfunction in the elderly^2^^,^^23^.

Depression is a common symptom in the elderly with hearing loss because of the daily life limitations that such loss brings about^6^^,^^24^^,^^25^. In the present study, 15% of the sample had GDS abnormalities, which confirms the importance of assessing depression aspects, even in the elderly without complaints^26^.

The P300 latency results show that both the right-side afferent pathways as well as the left side ones are in agreement with the values stipulated by the specialized literature, which varied between 300 and 450 ms in elderly with hearing loss^1^^,^^13^^,^^27^, ^28^, ^29^, ^30^. Moreover, we noticed that there was a strong agreement between the latencies of the two ears.

The education variable did not correlate with mean P300 latency values. The study from Schiff et al.^30^ stated that education did not influence P300 latency - in agreement with data from the present study. Nonetheless, most studies with the P300 have paired samples in relation to education, making it impossible to check its influence on P300 latency.

The P300 latency values found in this study showed a positive correlation with age, in other words, age increase caused an increase in P300 latency. Analyzing studies in the specialized literature, we found a consensus that age impacts P300 latency^1^^,^^27^^,^^29^, ^30^, ^31^, ^32^, ^33^, ^34^, ^35^. Aging, added to hearing loss can bring about changes to the central auditory system, which causes a reduction in the signal processing speed^33^.

In our study we did not see any influence of the degree of hearing loss in the P300 latency. Therefore, peripheral hearing loss does not preclude the use of this procedure. Musiek & Geurkink^36^, Hall^37^ and Reis & Iorio^9^ reported that the P300 is not affected by hearing loss, as long as the individual can perceive the stimulus.

Although the P300 latency showed larger mean values in individuals with cognitive abnormalities when compared to elderly without cognitive abnormalities, such difference was not significant. Many studies in the literature showed a significant correlation between the P300 and performance in cognitive tests^27^^,^^29^^,^^34^^,^^38^. We must stress that the elderly who participated in the current study were not diagnosed with dementia. Thus, these studies produced results which cannot be directly challenged by those found in the present study.

We know that in cases of mild cognitive decline - which frequently precedes Alzheimer's disease, usually there is an increase in P300 latency, when compared to controls of the same age; however, absolute values are usually within the criteria of two standard deviations above the mean. Notwithstanding, when dementia is confirmed, the P300 latency values are off the normality range.

The increase in latency can be seen in subjects with depression, since depression symptoms may worsen the cognitive performance of the elderly^7^^,^^39^. Notwithstanding, in this sample there was no significant relationship between the GDS results and the P300 latency, in other words, psychological factors did not influence this potential's result.

In short, we noticed that the electrophysiological assessment yielded mean latency values which were expected for the age range of the elderly. Age increase brought about an increase in P300 latency. Going against the initial hypothesis of the present study, the other factors investigated: cognitive performance, depression symptoms, degree of hearing loss and educational level, did not influence the mean P300 latency values. Thus, despite the diversity of diseases and dysfunctions presented by the elderly, which could influence the P300 cognitive potential, such potential proved to be stable, and it can be safely used in clinical and scientific settings in the elderly with hearing loss.

## CONCLUSION

The advance of age caused an increase in P300 latency. Cognitive performance and depression symptoms, assessed by the MME, ADAS-Cog and GDS did not influence the P300 results in this population.
